# International clinician perspectives on pandemic-associated stress in supporting people with intellectual and developmental disabilities

**DOI:** 10.1192/bjo.2022.49

**Published:** 2022-04-18

**Authors:** Joshua Howkins, Angela Hassiotis, Elspeth Bradley, Andrew Levitas, Tanja Sappok, Amanda Sinai, Anupam Thakur, Rohit Shankar

**Affiliations:** Department of Public Health, NHS Grampian, UK; Division of Psychiatry, University College London, UK; Department of Psychiatry, University of Toronto, Canada; Rowan University, USA; Department for Mental Health, Charité University Hospital, Germany; Department of Psychiatry, Mayanei Hayeshua Medical Center, Israel; Department of Psychiatry, University of Toronto, Canada; Cornwall Partnership NHS Foundation Trust, UK; and Cornwall Intellectual Disability Equitable Research (CIDER), University of Plymouth Peninsula School of Medicine, UK

**Keywords:** Intellectual disability, developmental disorders, psychosocial interventions, patients, transcultural psychiatry

## Abstract

**Background:**

People living with intellectual and developmental disabilities (IDD) have suffered disproportionately in health outcomes and general well-being during the COVID-19 pandemic. There is emerging evidence of increased psychological distress. Increased strain has also fallen on clinicians managing the psychological needs of people with IDD, in the context of learning new technologies, staff shortages, reduced services and paused training opportunities.

**Aims:**

To examine clinicians’ experiences of patient care, clinical management and the impact of care delivery.

**Method:**

A mixed fixed-response and free-text survey comprising 28 questions covering four areas (responder demographics, clinical practice, changes to local services and clinician experiences) was developed, using the STROBE guidance. It was disseminated through an exponential snowballing technique to clinicians in seven high-income countries. Quantitative data were analysed and presented with Microsoft Excel. Qualitative data were coded and thematically analysed, and presented with in-text quotations.

**Results:**

There were 139 respondents, mostly senior physicians (71%). Two-thirds reported over 10 years working in the field. Quantitative findings include increased clinician stress (77%), referrals (53%), patient distress presentations (>70%), patient isolation (73%) and carer burden (89%), and reduced patient participation in daily activities (86%). A third reported increased psychotropic prescribing. Qualitative analysis outlined changes to clinical practice, particularly the emergence and impact of telehealth.

**Conclusions:**

In the countries surveyed, the pandemic has not only had a significant impact on people with IDD, but also their carers and clinicians. A proactive, holistic international response is needed in preparedness for future public health emergencies.

People living with intellectual and developmental disabilities (IDD) have suffered disproportionately during the COVID-19 pandemic.^1–3^ They have been at increased risk of hospital admission and death,^4^ and many have missed out on regular activities as services have been paused during imposed lockdowns.^5,6^ Family members and carers have had to step in to provide additional support even while struggling with their own anxieties about the pandemic, all of which may have had an impact on this vulnerable population.^7–10^ In times of distress and challenging behaviours, this population may experience overprescribing, particularly of psychotropic medications.^11–14^

The priority concerns for people with IDD in the UK during the pandemic have previously been explored through stakeholder engagement.^15^ The wide-ranging 28 statements identified as important to this vulnerable population cover mental health, including overmedication, carer strain and diagnostic overshadowing; physical health, including lack of access to services and clinical review; and social circumstances, including isolation, placement breakdown and risk of neglect. Although there have been similar national surveys in different countries, no comparative or international data is available. Little is known systematically on what the concerns are for similar high-income countries. This paper looks to explore clinicians’ perspectives, working in different health and social care systems, the challenges faced and lessons learnt to date from the pandemic, with a view to fostering shared learning on the concerns for this vulnerable population and carer and clinical stakeholders.

## Aims

We aimed to examine the experiences of mental health clinicians working with people with IDD during the COVID-19 pandemic in high-income countries.

We focus on two aspects: understanding the impact of the pandemic on patient care and clinical management, and understanding the impact of the pandemic on clinicians’ delivery of care.

## Method

### Survey development and dissemination

The survey was constructed by using the findings from a study exploring the priority concerns of representative organisations in the UK as a starting point.^15^ It focused on high-income countries to provide generalisable results. A working group of psychiatrists linked to different professional networks (e.g. European Association for Mental Health and Intellectual Disabilities, American Academy of Developmental Medicine and Dentistry, etc.) in seven different countries was convened, seeking collaboration on survey design and dissemination. The expert opinion of the working group allowed development of the survey content, requiring multiple iterations of the core text until consensus was achieved. Questions and phrasing were adapted to allow for generalisability and international variation in service provision, funding and structure.

The survey comprised a mixture of fixed-response and open answers across four sections with 28 questions total. The first section explored demographics and working setting, section two considered clinical practice, section three concerned changes to local services and section four focused on clinician needs and experiences.

Translations of the survey were provided, as required, developed locally by the representative of the country on the working group. These were checked before dissemination and again, after translating responses back into English. Any quotes used in the main paper and in the Supplementary material available at https://doi.org/10.1192/bjo.2022.49, are best-matched translations, with thematic match to the original closely checked by native speakers.

The survey was undertaken online on Google Forms for Windows 10 (see https://www.google.co.uk/intl/en-GB/forms/about/), with approximately 8–10 min given for completion. This was felt to be the optimum time to balance response engagement and gain the minimum required information to draw meaningful conclusions on the key identified areas. The survey was available online for 6 months, from 8 February to 9 August 2021.

Clinicians working with people with IDD were invited to complete an online cross-sectional survey comprising mixed quantitative and qualitative (free-text) answer options. The survey template is provided in Supplementary Appendix 1. In the introductory section of the survey, it was specified that this survey was aimed at clinicians working primarily with people with IDD, along with a question to check the same.

The survey used an exponential and non-discriminatory snowballing technique. This involves commencing with key personal contacts in professional organisations of the authors in different participating countries, and requesting then to forward the request and link within their own professional networks. This should be considered non-probability sampling, Reminders to encourage participation were sent to the working group at monthly intervals.

### Ethics and governance

All participants were advised at the start of the study that participation was voluntary and that their replies, if they chose to participate, would be anonymised and analysed. No participant identifier data was collected. Data was pooled before analysis. Further, it was to a professional participant group where consent was implicit by participation. It was specified that informed consent would be presumed if participants submitted the survey. Investigators obtained local approvals as necessary, and were responsible for local oversight.

The authors assert that all procedures contributing to this work comply with the ethical standards of the relevant national and institutional committees on human experimentation and with the Helsinki Declaration of 1975, as revised in 2008. All procedures involving human patients were approved by the University of Toronto Research Ethics Board Protocol (protocol number 40791).

Ethics advice at various levels across the different participating organisations was collected. Ethics approval was obtained from University of Toronto Research Ethics Board (protocol number 40791). Investigators also obtained local permission after consulting their local ethics board as necessary. In North America, the project was promoted to the medical membership of the American Academy of Developmental Medicine and Dentistry. Before promotion, the project was reviewed by its research committee. The need for ethics approval in Israel was discussed with the hospital ethics (Helsinki) committee and, because of the nature of the research, was not found to be required. Similarly, Germany and other participating countries did not need ethics on consultation with their local boards.

### Analysis

Anonymised responses to the survey were downloaded into Microsoft Excel 2019 for Windows, then transferred to SPSS version 25 for Windows for data cleaning, coding and analysis. As most of the fixed-response variables were categorical or ordinal, analysis consisted mainly of frequencies and cross tabulations.

Free-text replies were assimilated by the first author. Authors J.H. and A.H. read the free-text responses to become familiar with topics, and began preliminary coding for thematic analysis. Coding developed with additional reading, analysis and further interobserver discussion. The two authors compared coding and noted any differences. Differences were resolved by discussion and agreement on the final coding frame. Author R.S. provided support to resolve any outstanding differences. All raw data and associated themes were grouped for analysis. Themes were discussed and presented based on their frequency weighting in responses.

As country-specific response weightings were heavily skewed, the authors were not able to comment on generalised differences between countries, as the small number of respondents from some would have introduced excessive bias.

## Results

### Quantitative responses

#### Study population

Responses were provided by 139 clinicians spread across seven countries: Germany (*n* = 54, 38.8%), Canada (*n* = 32, 23%), USA (*n* = 31, 22.3%), Switzerland (*n* = 12, 8.6%), Austria (*n* = 6, 4.3%), Israel (*n* = 3, 2.2%) and Argentina (*n* = 1, 0.7%).

Nearly three-quarters (*n* = 99, 71.2%) of the respondents declared themselves as senior or higher/advanced level psychiatrists, with the rest being junior psychiatrists or other, including nurses and other healthcare professionals (Table 1).

**Table 1:** Professional grade of respondents, including missing data (data not provided by respondents)

Professional grade	Number of respondents (% total)
Senior level physician	89 (64)
Higher/advanced trainee physician	10 (7.2)
Junior trainee physician	1 (0.7)
Advanced practice nurse	3 (2.2)
Other senior healthcare professional	13 (9.4)
Other non-senior healthcare professional	4 (2.9)
Missing^a^	19 (13.6)

a. ‘Missing’ data-set here and below accounts for any non-selections for each question from the 139 participants.

Of 137 participants who quantified how much of their workload is with people with IDD, nearly two-thirds (*n* = 87, 62.7%) worked primarily (>50%) with people with IDD. Twenty-four (17.3%) spent 25–50% of their time working with people with IDD, whereas only 26 (18.7%) spent <25% of their time working with people with IDD. Two-thirds of respondents (*n* = 92, 66%) had been working with people with IDD for >10 years.

#### Effects of the pandemic on clinician working patterns

Just over three-quarters of respondents (*n* = 107, 77%) noted feeling increased stress since the pandemic, compared with pre-pandemic levels. This spread was relatively uniform among participating countries. Of the top responding countries, 26 (87%) participants from the USA noted increased stress, as did 24 (75%) participants from Canada and 40 (74.1%) participants from Germany. Most clinicians experienced an increase in referrals of people with IDD (*n* = 74, 53.2%). However, a significant minority of 25% (*n* = 35) noted a decrease in their referral pattern (Table 2). Considering time spent in consultation, nearly three-quarters (*n* = 101, 72.7%) remarked spending more time in consultation, with 11 respondents (7.9%) increasing their time in consultation by >50% (Table 3).

**Table 2:** Change in referral pattern for people with intellectual and developmental disabilities

Change in referral pattern compared with pre-pandemic patterns	*n* (%)
Increase	
>50%	9 (6.5)
25–50%	14 (10.1)
10–25%	31 (22.3)
0–10%	20 (14.4)
Decrease	
0–10%	8 (5.8)
10–25%	21 (15.1)
25–50%	6 (4.3)
>50%	0 (0)
Not applicable/missing	30 (21.6)

**Table 3:** Change in time spent in consultation with people with intellectual and developmental disabilities compared with pre-pandemic consultations

Change in time spent in consultation	*n* (%)
Increase	
>50%	11 (7.9)
25–50%	20 (14.4)
10–25%	35 (12.2)
0–10%	35 (12.2)
Decrease	
0–10%	3 (2.2)
10–25%	7 (5)
25–50%	0 (0)
>50%	0 (0)
Not applicable/missing	28 (20.1)

#### Impact of the pandemic on patient mental and physical health

Regarding areas of work affected by the pandemic (Table 4), over three-quarters (*n* = 108, 77.7%) noted an increase in new challenging behaviours observed. A similar proportion (*n* = 102, 73.3%) also stated that they had observed an increase in previously existing challenging behaviours, and over six-sevenths (*n* = 121, 87%) also noted an increase in referrals for new-onset emotional distress, with the majority (*n* = 109, 78.4%) observing an increase in previously existing emotional distress observed in their patients. Most respondents (*n* = 119, 85.6%) observed reduced patient participation in activities, with nearly three-quarters (*n* = 101, 72.7%) reporting patient feelings of isolation. Just over half of the respondents, (*n* = 73, 52.5%) reported an increase in their concern for the physical health of their patients (Table 4). Four out of five participants (*n* = 111, 79.8%) considered their patients increasingly unable to cope with changes to staff or routine (Table 4).

**Table 4:** How respondents feel the pandemic has changed the noted areas in their practice with people with intellectual and developmental disabilities

Criteria	Scale of change	*n* (%)
New challenging behaviours		
	Significant increase	33 (23.7)
	Slight increase	75 (54)
	No change	23 (16.5)
	Slight decrease	6 (4.3)
	Significant decrease	0 (0)
	Missing data	2 (1.4)
Existing challenging behaviours		
	Significant increase	28 (20.1)
	Slight increase	74 (53.2)
	No change	28 (20.1)
	Slight decrease	5 (3.6)
	Significant decrease	2 (1.4)
	Missing data	2 (1.4)
New emotional distress		
	Significant increase	53 (38.1)
	Slight increase	68 (48.9)
	No change	10 (7.2)
	Slight decrease	4 (2.9)
	Significant decrease	1 (0.7)
	Missing data	3 (2.2)
Existing emotional distress		
	Significant increase	33 (23.7)
	Slight increase	76 (54.7)
	No change	21 (15.1)
	Slight decrease	1 (0.7)
	Significant decrease	2 (1.4)
	Missing data	6 (4.3)
Patient participation in activities		
	Significant increase	8 (5.8)
	Slight increase	4 (2.9)
	No change	6 (4.3)
	Slight decrease	17 (12.2)
	Significant decrease	102 (73.4)
	Missing data	2 (1.4)
Patient feelings of isolation		
	Significant increase	61 (43.9)
	Slight increase	40 (28.8)
	No change	12 (8.6)
	Slight decrease	8 (5.8)
	Significant decrease	15 (10.8)
	Missing data	3 (2.2)
Physical health concerns		
	Significant increase	12 (8.6)
	Slight increase	61 (43.9)
	No change	55 (39.6)
	Slight decrease	7 (5)
	Significant decrease	2 (1.4)
	Missing data	2 (1.4)
Carer burden and strain		
	Significant increase	77 (55.4)
	Slight increase	46 (33.1)
	No change	8 (5.8)
	Slight decrease	1 (0.7)
	Significant decrease	3 (2.2)
	Missing data	4 (2.9)
Placement of home breakdown		
	Significant increase	10 (7.2)
	Slight increase	38 (27.3)
	No change	77 (55.4)
	Slight decrease	5 (3.6)
	Significant decrease	4 (2.9)
	Missing data	5 (3.6)
Closure or restriction of day or respite centres		
	Significant increase	102 (73.4)
	Slight increase	18 (12.9)
	No change	3 (2.2)
	Slight decrease	1 (0.7)
	Significant decrease	11 (7.9)
	Missing data	4 (2.9)
Changes in daily/weekly routines		
	Significant increase	94 (67.6)
	Slight increase	27 (19.4)
	No change	4 (2.9)
	Slight decrease	3 (2.2)
	Significant decrease	8 (5.8)
	Missing data	3 (2.2)
Person with intellectual and developmental disabilities unable to cope with changes to staff/routines		
	Significant increase	38 (27.4)
	Slight increase	73 (52.5)
	No change	21 (15.1)
	Slight decrease	1 (0.7)
	Significant decrease	2 (1.4)
	Missing data	4 (2.9)
Availability of wider clinical or care team support		
	Significant increase	11 (7.9)
	Slight increase	24 (17.3)
	No change	32 (23)
	Slight decrease	38 (27.3)
	Significant decrease	29 (20.9)
	Missing data	5 (3.6)
Difficulty accessing primary care		
	Significant increase	19 (13.7)
	Slight increase	49 (35.3)
	No change	48 (34.5)
	Slight decrease	12 (8.6)
	Significant decrease	7 (5)
	Missing data	4 (2.9)
Difficulty accessing specialist care		
	Significant increase	24 (17.3)
	Slight increase	53 (38.1)
	No change	38 (27.3)
	Slight decrease	9 (6.5)
	Significant decrease	12 (8.6)
	Missing data	3 (2.2)
Pressure/difficulty securing in-patient or emergency resources		
	Significant increase	25 (18)
	Slight increase	53 (38.1)
	No change	48 (34.5)
	Slight decrease	3 (2.2)
	Significant decrease	5 (3.6)
	Missing data	5 (3.6)

With regards to the impact that restrictions on social, educational or vocational activities had on certain domains, just over half (*n* = 73, 52.5%) noted an increase in psychiatric conditions, two-thirds (*n* = 92, 66.2%) noted a specific increase in emotional distress and two-thirds (*n* = 92, 66.3%) noted an increase in challenging behaviours. Most respondents noted no change observed in patients’ physical health (*n* = 100). Over seven-eighths (*n* = 123, 88.5%) observed increased carer burden and strain.

#### Impact of the pandemic on treatment and support

Considering management methods available to clinicians, just over a third (*n* = 48, 34.5%) of respondents noted an increase in their overall prescribing practices since the pandemic, although 56.1% (*n* = 78) noted no change. However, when considering specific prescribing domains (Table 5), 46.8% (*n* = 65) reported an increased use of antipsychotics, 43.9% (*n* = 61) reported increased use of antidepressants, 30.2% (*n* = 42) reported increased use of mood stabilisers and 25.2% (*n* = 35) reported increased use of benzodiazepines. Nearly half also reported (*n* = 64, 46%) more use of medications for physical ill health. Just over half of the respondents reported not using outcome measures or scoring systems to monitor for changes influenced by psychotropic prescribing (*n* = 73, 52.5%).

**Table 5:** How respondents’ prescribing practices have changed because of pandemic-related restrictions

Criteria	Scale of change	*n* (%)
Antipsychotic prescribing		
	Increased	65 (46.8)
	No change	44 (31.7)
	Decreased	1 (0.7)
	Missing data	29 (20.9)
Antidepressant prescribing		
	Increased	61 (43.9)
	No change	46 (33.1)
	Decreased	0 (0)
	Missing data	32 (23)
Mood stabiliser prescribing		
	Increased	42 (30.2)
	No change	64 (46)
	Decreased	1 (0.7)
	Missing data	32 (77)
Benzodiazepine prescribing		
	Increased	35 (25.2)
	No change	70 (50.4)
	Decreased	3 (2.2)
	Missing data	31 (22.3)
Stimulant prescribing		
	Increased	14 (10.1)
	No change	87 (62.6)
	Decreased	5 (3.6)
	Missing data	33 (23.7)
Pain medication prescribing		
	Increased	10 (7.2)
	No change	97 (69.8)
	Decreased	0 (0)
	Missing data	32 (23)
Physical health medication prescribing		
	Increased	64 (46)
	No change	41 (29.5)
	Decreased	3 (2.2)
	Missing data	31 (22.3)

#### Impact of the pandemic on service organisation

Most clinicians had been involved in the local implementation of service changes in response to the pandemic (*n* = 89, 64%). Nearly two-thirds (*n* = 90, 64.7%) reported some subjective positivity toward how successful their area had been in adapting to their proposed changes, and most (*n* = 102, 73.4%) had provided information to their patients relating to the service changes experienced.

### Free-text (qualitative) responses

Free-text responses to ‘open’ survey questions were collected to gather information on personal experiences of responding clinicians. These are available in full in Supplementary Appendix 2. Key themes that developed were those denoting practice-based challenges and adaptations (relating to changes in how respondents had practised during the pandemic), intervention-based adaptations (relating to what was done or offered within that practice) and responses relating to resource considerations, both during the pandemic and when considering the future.

#### Practice-based adaptations

A major subtheme within the changes to clinicians’ practice was increased use of technology to facilitate remote working, often considered in the raw data as telehealth.

Generally, responses were positive for clinicians, ‘I have found virtual assessments more useful than I would ever have imagined’, and when clinicians considered their patients, ‘It helps make appointments easier to attend for some of my patients’.

However, challenges were noted within this, including issues of access and education: ‘lack of access to telehealth technologies’, ‘difficulty with internet or device’ or ‘technology not always available to clients and/or caregivers; knowledge lacking’.

#### Intervention-based adaptations

Intervention-themed responses highlighted a potential increase in prescribing patterns, consistent with what is seen in the fixed-response questions. However, the implicit future requirement to titrate these back down once wider opportunities reopened was often noted. Respondents highlighted the need to ‘laboriously reduce the medication again over a long period of time’ and the potential future need for ‘deprescribing programmes’.

In addition, clinicians often remarked on an increase in referral patterns to psychological services. ‘Psychotherapy’ and ‘psychoeducation’ were frequently mentioned when considering newly initiated interventions. Often, this was mentioned in relation to disruption of daily routines, lack of replacement recreational activities or lack of socialisation.

The increased referral to psychotherapeutic therapies matched a general increased reliance on the wider multidisciplinary team. Some respondents felt this enhanced ‘interdisciplinary collaboration’ and ‘more productive’ teamwork, although the same issues with access and administration mentioned above were also frequently cited.

#### Resources

Increased administrative workload was the key subtheme for resource-focused responses. This was noted in terms of the shift to telemedicine and from the unavoidable ‘staffing problems’, which often considered both a lack of staff and lack of appropriate training. Clinicians reported taking on greater responsibility to make up for this shortfall.

Worryingly, clinicians noted concerns over stress and burnout, remarking ‘We are hanging in there but with chronic fatigue’.

The changes in service delivery influenced statements about plans for future service changes that would offer ‘better organisation and integration of services’.

Much call was noted for ‘more support both financially and emotionally’ to deal with the expected continued increased demand on services and backlogs, but this may pragmatically be tempered by concerns of likely ‘budget cuts/funding restrictions’. One respondent summarised their concerns as ‘still a neglected population with worse set back in even basic health, mental health and supportive care’.

## Discussion

This is the first survey of its kind to consider the experiences and views of clinicians working primarily with people with IDD during the pandemic in high-income countries, and how this challenging circumstance has affected practice and care. Importantly, it provides a wide perspective, not limited to one healthcare system. It is interesting to note the similarities of experience provided by respondents from countries practising in diverse settings.

Although the change in working patterns may have been expected, it is notable how substantially these have affected clinicians, both in terms of time spent at work and stress felt. The stress felt by people with IDD is shown vividly, with a worrying increase in emotional and physical distress and behaviours that challenge. The impact of restrictions on this vulnerable group is laid bare, with reduced engagement and isolation occurring in context of changes to daily routine and curtailment of engagement in social, educational or vocational activities.

The impact of restrictions may have been a cause for the reduction in referrals in a significant minority of respondents (25%). Restrictions might have provided stability, consistency and predictability to a select group, such as those with autism, who face significant anxiety during ‘normal times’ because of the environmental and social unpredictability of our society. Equally, it could have led to social withdrawal and a loss of contact from services. This intimates the complicated nature of need and management of this group.

The overarching feeling from considering the responses is that health and healthcare have changed for the worse for those with IDD, their carers and the professionals working with them. Many free-text responses considered an irreversible shift in their practice. Some of this, particularly a shift toward telecommunications, may be considered beneficial, and indeed may have served to speed up positive transition that has been long overdue. However, the pace of change has left many feeling overwhelmed or unsafe, and raises important concerns about equity of access for people with IDD. The financial cost of widespread uptake of telehealth, both institutional and individual, remains unquantifiable, but is likely to be significant. The increased productivity and efficiency, and the relief from travel that patients may experience, may eventually tip the balance in favour of this upheaval.

Other changes do not appear so welcome, particularly the increase in prescribing patterns, which agrees with previously published data in this population.^11–14^ It will be a long and complicated journey to work with patients toward reducing these medications. It is important that psychotherapeutic support is brought back in line with the national policy of all participating countries to stop and reduce continued harm of inappropriate psychotropic prescribing.

Observance of clinician and carer stress in responses is frightening, but not unexpected. Supporting individuals with IDD is complex even in ‘normal times’. The highlighted impact on activities, roles and engagement has possibly allowed a domino effect of stress and burnout onto families and clinicians. Efforts will be required to improve resource allocation and training, and clear backlogs, when pandemic-affected practices start recovering. These efforts to reduce the digital divide by proper accommodation can help to reduce inequities in access to healthcare for people with IDD. Care should be taken to continue the increased interprofessional working reported by our respondents, and to support implementation of evidenced best practices and strategies for this population while ensuring reduction of siloed practicing. The study suggests a call for structural service changes is required. This change needs to be in the form of renewed focus on staff welfare and training, continuing integration and collaboration between services, and a combined approach to digital and in-person provision where suitable.

### Limitations

This paper contains several limitations owing to its opportunistic sampling frame, focus on high-income countries and with an unequal spread even between these countries. It is presented as a pragmatic attempt to stimulate discussion. In-depth demographics were not requested, so the reach within minority or marginalised groups is unclear. It is not to be considered generalisable without further consideration. The responses represent only a snapshot of clinicians’ contemporary feelings on this subject. With the ever-changing professional and social context in which clinicians currently operate, it must be acknowledged that responses and experience may change dramatically with time, and this paper can only be seen as presenting a snapshot of opinions in context of the time at which it was taken. Responder bias is likely to be present, with those with negative feelings and experiences more likely to respond and give detailed answers. The untrialled, unvalidated survey is likely to present a measurement bias. However, we present the full raw data in mitigation of the inherent observer bias. The survey findings could be considered broadly generalisable to high-income countries, given its reach. However, there is a possibility that important vulnerable subpopulations in these countries, such as Black and Asian ethnic minorities who have been disproportionately affected, have not been suitably represented.

### Implications for clinical practice

Careful work will be required to encourage, permit and empower people with IDD to recover their normal lifestyles following the pandemic. This will need to go hand in glove with weaning any additional psychotropic burden necessitated by the challenges of pandemic-related restrictions and the negative impact on mental health and challenging behaviours. Clinicians must be mindful of the increased morbidity across this vulnerable group, maintain a holistic approach to understanding the impact of the pandemic on the individual and consider the range of presentations different individuals may present with, including as-yet-unknown presentations of ‘long COVID’. Clinicians must be conscious of the bi-directional interplay of health and social care, and the already overburdened and stretched areas of each. Time will be required to catch up on missed training and supervision for clinicians. There is also the issue of addressing clinician trauma and protecting the existing workforce while helping replenish it.

It worth noting that although this is the first study internationally focused on intellectual disability, there exists literature on similar concerns for other vulnerable populations, such as those with serious mental illness or dementia.^16,17,18,19^ Common clinical themes are exacerbation of loneliness, isolation, social disruption and overall stress and anxiety. Similar to our study findings, there are concerns regarding vulnerable populations being left behind, particularly those struggling to engage in remote care. Thus, these shared affinities could form part of a common clinical pathway for people with intellectual disability with other similar vulnerable groups.

### Implications for research to inform policy

Although the pandemic has undoubtedly influenced health and social outcomes globally, there needs to be recognition of the unequal harm borne by this vulnerable group. The pandemic has negatively affected research, training and education in mental health services in general, and intellectual disability services in particular.^20^ Thought needs to be given to a systems approach to research, to understand the less-often recognised burden of stress in carer and clinicians supporting this group. Most additional burden has fallen on carers, and in-depth analysis of methods to support re-engagement, holistic care and carer support will be required. An international holistic policy considering all of these aspects underpinned by evidence-based research might optimise a more comprehensive understanding and appreciation of the issues, and guide a robust approach to future service provision and development.

## Data Availability

The data that support the findings of this study are available from the corresponding author, R.S., upon reasonable request.

## Supplementary material

For supplementary material accompanying this paper visit http://doi.org/10.1192/bjo.2022.49. click here to view supplementary material
