# Supplementary material for: International clinician perspectives on pandemic-associated stress in supporting people with intellectual and developmental disabilities
Source: BJPsych Open. 2022 Apr 18;8(3):e84. doi: 10.1192/bjo.2022.49 (PMC9059730; doi:10.1192/bjo.2022.49)
Supplement: Supplementary file 1 [file bjosup.zip › S2056472422000497sup002.docx]

Supplementary information 2 - Qualitative responses (raw text)

What other interventions have you considered or initiated?

N/A

1 on 1 interviews

Behavioral supports

Behavior Support Plans

Counseling, ABA

collaborative work with teams

Na

Behavior analysis

Practice Dentistry

Information on COVID and mental health from variety of sources. Much more telehealth

Working with psychologists to be able to team up with and modify behavior plans

Behavioral interventions

The usual: Review of school placement where appropriate, advice to parents/teachers, regular parental guidance, individual psychotherapy, behaviour therapy, i.e. no change.

one to one driving time. Getting residents out of the house without leaving the car. ( special lunch ... etc. )

Zoom contacts with psychology and behavioral therapists

Referral to short term behavioral health consultant remotely

DBT informed Skills system therapy

increased focus on metabolic health

Counseling

DBT, CBT

Social and environmental adjustments rather than medications. For example, structured adherence to new routines, preferred activity substitutions, exercise, nutritional monitoring

none

Much more in-person support from preferred direct support professionals

Cognitive Behavioral Therapy and Dialectical Behavioral Therapy

x

None

try to counsel caregivers on how to help patients and be supportive of their efforts when ther are shutdown or limits to services

Behavioral, structural, use of virtual

telehealth has majorly taken off in my practice/ many more team meetings on zoom

more direct contact with schools to facilitate support /understanding of challenging behaviour

Environmental efforts are maxed , this helps a some. Things we normally cannot get paid for are being paid for but when you cannot see your parents or siblings or get out for a walk swings and trampolines do not substitute for family. Access to paraprofessionals who know how to facilitated communication and socialization are still very rare in this province.

virtual care, increased online groupwork for people with NDD, carer support groups, capacity building initiatives in the community

BT, OT

Behavioural modification

Behavioral therapy, counselling services through Surrey Place

Helping families access more community supports; outdoor activity

Virtual care; offered to help with vaccine roll out in group homes; no response from OPH

tele medicine

mindfulness, acceptance-based therapy

data collection with environmental modifications as indicated

We have a psychiatrist work with us we discuss intervention,sometimes restrains used.

Attempt to get BT, OT, SW involvement as needed

We have a psychiatrist work with us and have developed a protocol.

behavioural therapy

virtual counselling; more frequent follow ups

Social services support

None

N/A

rTMS, ECT, medical cannabis

Nothing different except some online groups

no

psychotherapy

Psychotherapy, inpatient admissions

Clarification / diagnostics and advice; if necessary (psycho-) therapeutic treatment

more advice and medication for falling asleep and staying asleep

Improvement of the daily structure offered by residential carers

More frequent and more intensive psycho-education of the care system

Occupational therapy, psychotherapy, more 1: 1 care, advise to structure the day through the caring environment with physical exercise, autism-specific treatment, coaching of carers with regard to autism-specific needs

Psychoeducation, autism-specific interventions; Behavior therapy

Interdisciplinary collaboration, systemic and individual psychotherapy, also trauma-specific, psychoeducation in an outpatient setting.

Observation sheets

Consulting, systemic concepts, team supervision

Interdisciplinary zoom conferences with the supporting team

Prescription of occupational therapy, increased use of individual support in everyday life

I hope for proper feedback from the residential groups regarding Medication, otherwise only frequent walking was established

Structured daily routine, external activities

More common 1: 1 situations

Therapeutic interventions (individual discussions)

Crisis plan, behavior observation sheet, telephone queries

Re-establishing day structure and activities, seeking for alternative activities

Improvement of day-structuring measures in the home facilities

Behavior Therapy / Anxiety Treatment

Inpatient diagnostics and therapy

psychotherapeutic support

psychological interventions, recreational opportunities

Organization of everyday life through employment offers taking into account the safety / hygiene measures

Anamnesis from carers

more parental work and case conferences with caregivers

Security concepts

Consulting of legal guardians

A lot of talks with relatives

I am an orthodontist and therefore I do not prescribe any medication, but I learn a lot from discussions with patients about increased medication requirements. Since the pandemic, I have been taking significantly more time for these patients who are suffering much more severely under the circumstances, some of whom cannot understand, e.g. why they have to restrict contacts or e.g. for my deaf patients who can no longer easily read their lips.

Structural changes in the facilities, individual support

Educators, behavioural therapists and other employees

More physical / occupational therapy

More frequent visits to the facility

Special educational support

Psychological specialist service

Youth welfare measures, outpatient psychotherapy, rehabilitation measures, integration measures

conversations

Psychotherapy, individual care, home leave for relatives, ..

Conversations, Psychotherapy

increased specialist advice

psychological counseling

Training of staff in dealing with emotional dysregulation, self-aggression; a lot of work on validation of the team; more intensive pycho-education of the referring institutions

Systemic multi-professional interventions

Medical, psychosocial and behavioural interventions are discussed with the teams and implemented if necessary

Psychotherapeutic discussions

inpatient clarification

behavioural therapy

What barriers have you encountered in implementing these interventions?

Staff stressors

Resistance

Access to behaviorists

Stress among caregivers with less adherence to protocols.

Not enough access to providers

N/A

accessibility, internet challenges

Na

Availability

Practice Dentistry

In my state, public insurance (Medicaid) not paying as well for telehealth

Staffing problems, time constraints, caregiver burnout

Funding from medicare and medicaid

Unavailability of behaviour therapy in my team. This is not a new problem.

lack of interest or energy to get out of the house. Lack of interest due to lack of stimulation

Family access to computers, internet service and comfort with telemedicine

Waiting period has increased. This is not her area of expertise.

All by tele health so difficulty with internet or device

need for increased motivational interventions when individuals have severely limited activities and choose to spend their money on food and tobacco while not getting exercise

Challenges with isolation and limited telehealth resources

Lack of access to telehealth technologies, lack of privacy in residential settings to receive services via telehealth

Staff morale is lower than patient morale, because they are more aware of the gravity of the situation and they have lost more autonomy than our patients due to the pandemic.

staff time

staff shortages

Ttelehealth consults versus in office visits

x

None

When patients residences are not allowed to bring pts to clinic due to temporary lockdown and they expect phone assessment not even video assessment

Poor WiFi, limited iPad or computer, group home restricted

Less day programs have led to increase agitation in some patients

school closures / school staff stressors

Access to family because of lock down, access to knowledgeable paraprofessionals and consistency in staff training to put programming in place to off set lockdown. It is really challenging staff on multiple leaves. Not their fault because there are not enough of them, not enough professional development and they are all stressed as well.

Access to virtual care has been difficult for some people due to difficulties in use of technology. Privacy issues were cited in some cases.

virtual care limits the scope and extents of services

Not enough professionals or no access

Lack of resources

Most formal supports are not taking new referrals during pandemic, but churches and other informal supports can still be helpful.

Technology not always available to clients and /or caregivers; knowledge lacking

technical, cultural

lack of patient and caregiver buy-in

Staff shortages - unable to collect data or implement plans

usually not because I talk to the family/caregiver,the reason and for limitted time use.

Lack of publicly funded resources

Resistence from family member.

virtual interventions

not enough time and increased need; kids out of school means less monitoring for kids in high risk social settings and has complicated risk/benefit decision making around prescribing or accessing programs

availability of specialists including dental

Lack of developmental services involved

Bureaucratic aspect is too slow

n/A

Guardian acceptance/consent

availability of online groups and the ability to access them by people with moderate/ severe ID

none

the usual

no capacities (to apply the recommendations)

the usual difficulties of working with people with developmental disabilities

none, great acceptance

Shortage of staff, untrained leasing staff

Low personnel resources in the residential institutions

Shortage of staff / fluctuation in residential home, for example, restriction of, for example, occupational therapy in residential homes due to access restrictions

reduced human resources due to lockdown

There is no adequate inpatient child and adolescent psychiatric facility for this patient group, even with no parent-child unit,

Lack of accompanying staff

Space shortage, distance problems

direct contact is partly missing

no secure contact options, as therapists in some facilities had no access (pandemic-related)

Little flexibility/more restrictions

personnel restriction

Resource problem

Infrastructure is only available to a limited extent, human resources are inadequate, and the adaptability of those affected is low in some cases

Group size very small for infection protection reasons, too few staff for small groups or individual care

Pandemic measures.

Decreased uptake of elective cases in clinics

Financing of on-site support, financing of telemedical care

no staff no money no rooms

More complex organization, as patients can be less actively involved

due to Covid more organizational difficulties (number of people, spatial conditions)

organizational difficulties in terms of legal restrictions for the employees

Personnel shortage in the facility (there is a lack of time and professional qualifications among the employees)

Lack of staff, dismissal psychologists

Time is always scarce ...

Hygiene standards to be observed

Comprehension problems

Therapy practices may or may not want to be in facilities

Lack of time

Lack of staff in residential facilities

Quarantine and isolation measures

Availability of contact persons (especially schools), scarcity of resources

Masks

Suspected C infection, overload ..

COVID-related

less availability

Scarce human resources

Communication in pandemic times, time expenditure

Rejection by legal guardian, caring employees or rarely clients, for fear of infection or subsequent stressful quarantine

Limitations under Corona

What changes do you foresee to your working practices post pandemic?

None

Need for more advanced care

Continued use of some proportion of telehealth

Increased sanitizing and PPE. Need for respirators. Inclusion of more telehealth appointments.

Expecting continued high need; some continued virtual services/telehealth

No changes

not sure

weight and movement, positive self image, routines

Continued use of more PPE

N95 s and PPE forever

Hope to be able to continue with more telehealth especially for our rural families

Telemed implementation

Unsure

Slight increase in telephone follow-up – from 0 to maybe 5% of my Workload.

increased community time for the residents, more visiting rights,

More telehealth for chronic condition management; necessity for identification and closure of care gaps, need for re-stabilization of chronic conditions after lesser monitoring during pandemic

Telehealth will continue.

Will continue with tele health

increased focus on physical health and preventive care

Decreased day program availability

Clientele not feeling safe or comfortable to return back for face to face services

Hope to continue to use virtual care for families with transportation difficulties /other barriers to accessing face to face services

Some days I consider retiring and other days restructuring.

increased virtual consultations

we need to find a new normal

Try to use telehealth more often. It helps make appointments easier to attend for some of my patients

Blending online and in-person work

Combination virtual and in person; increased demand

telemedicine. need for patient and family centered-care

more virtual care

more time managing difficult issues due to long wait for consultation

We may focus more on physical health,as it was taken less importence.

More virtual medicine

More Virtual Visits.

more outpatient work done virtually - in a positive way

Will continue some element of virtual care; in several cases close f/u has benefits for medication use and trouble shooting sfx. Still working to secure use of online standardized assessment forms (beyond the free tools)

more in person

Virtual options more

None

Social support for patients with NDD

More virtual care

would like to keep virtual care option

Yes, we use a lot of video conferencing to the benefit of patients. We spend less on travel and hence the team is more productive. Patients and care givers don't need to travel to come to clinic as before.

More use of telemedicine

no positive, at most exhaustion and dismissal

I expect "for the sake of simplicity" to keep online contacts to families with their children to the detriment of personal appointments.

Relaxation of one's own staff, increased demand for inpatient admission

Better predictability

Establishing the old day-to-day structures and, as a result, some problem behaviors resolve "by themselves", although some people with severe autism experience being overwhelmed by sheltered workshop visits, because in my opinion some benefit from the lockdown

Significant increase in secondary problems with regard to (re) integration into social systems (day care center, school; sometimes also family)

outreach work is becoming more and more established

Return to normal

higher demand for psychotherapeutic help

not clear

no

Unemployment - more family burden

uncomplicated contact possibilities eg with therapists, supervisors, "round table" possible again on site, relaxation for inpatients through regular visits from relatives (currently prohibited)

Even more compulsory digitization, video consultation hours to generate even more mountains of data, among other things, but a deterioration because of the shortage of physical doctor-patient contact, less closeness in relationships, less empathy, but more technical diagnostics, more fear of the doctors to contaminate with the desire to establish more distance

The care of my patients in the residential homes has further decoupled from the general practitioner care

no essential

more about telephone advice (possibly also video telephony)

I have no special expectations in this regard. Maybe more telemedicine is desired?

Laboriously reduce the medication again over a long period of time

More video consultation hours

more online / video visits

No

The number of patients increases as there is less fear of appointments away from home

Further development of telemedicine

no money

-

Increased rush of visitors, psychological stress from Corona processing

More opportunities to use modern means of communication, more flexibility in care and treatment structures P

slow stabilization of the patient

a lot online

Increase in patient numbers

Certain hygiene requirements will remain. Annual Covid vaccinations

more psychological / psychiatric offers

the increased need for hygiene, greater distance, etc. will remain

Those affected have to adjust to the environmental conditions again, fears / worries about another pandemic

only slow return to normal

That everything is sorted out again?

Challenging behaviours will persist

Normal life for the patient

more teleconferencing

the feeling of insecurity remains

Relief

no essential

The number of patients with psychiatric illness will increase. Increased challenging behaviors. Skilled workers shortage will worsen.

hope for improvement / stabilization of the situation

All in all, a facilitation of processes and improvement of communication with residential facilities

Changes of living groups, changes in the daily structure, more "young wild" cross-border commuters

No fundamental changes, possibly a slight increase in Myalgic encephalomyelitis/cronic fatigue syndrom in post-covid disease

Increase in service request

that unfavorable alienation of the Doctor and the patient persist due to fear of infection

frequent occurrence of advanced and complicated illnesses, catching up with preventive examinations

there will be even fewer people who want to work in medicine ?

What changes do you foresee for other patient services post pandemic?

Increase i telemedicine

Counseling

same

Challenges to resuming work in community settings - many support agencies have failed as a result of the pandemic. Financial impact.

Telehealth is here to stay

No changes

more televisits

self direct

Timing between appointments

More mental health referral resources

Budget cuts/ Funding reductuons.

More support both financially and emotionally

Probably other Serviceproviders will also do more virtual Follow– ups

not sure

More telehealth for chronic condition management; necessity for identification and closure of care gaps, need for re-stabilization of chronic conditions after lesser monitoring during pandemic; need for "catch-up" in vaccines, screenings

I'm concerned about the stresses from the economic downturn and coping with re-entry.

More tele health as well

ideally, better organization and integration of services

Decreased access

Greater focus on holistic responses.

none

I don't know what will happen

x

-

same as above with hybrid models of in person/zoom meetings

More virtual

Mauch more tlehealth

more online parent training options

Not sure there has been a lot of good advocacy in this province. The government knows the advocates are here and will hold their feet to the fire. Is that enough for change? Hard to say what will happen.

There will higher resource usage due to higher referral rates.

new order will emerge

I believe there will be a lot more online services. Unfortunately there will also be lengthy wait-times for in-person programming.

Huge backlog for accessing other specialists, as too many resources have focused exclusively on covid

increased demand

same

more virtual care

Long wait for consultation

Increase investigations/referals.

Not much.

similar changes

I hope tele health will remain; for some adolescents it has improved access to CBT without barriers of catchment area.

Still a neglected population with worse set back in even basic health, mental health and supportive care

None

Activities for those patients in addition the health care system

see above

no

More online offers

more Zoom Conferences

a hype for epidemic medicine, more controls, more separation

-

More layoffs in the sheltered workshops because patients also benefited from a lack of work in some cases

Reduction and focus of offers

possibly more online consultation hours

maximum financial stress for the clinics; Gain in modern communication technology

Improvement and expansion of mental health services

Healthcare offers will be reconsidered

Financial troubles

selective access / restrictions

Allocation to specialists is easier again, consultation services are available again on site.

more controls, more medical lists, less consideration of individual concerns, since the individual case eludes an assessment and evaluation by artificial intelligence and cost bearers

More government intervention

no essential

long waiting times

I have no special expectations in this regard either.

No

better and timely appointments

Savings

Greater demand

normal situations in the hospitals (do not just send necessary admissions back home)

Telemedicine is increasing

Increase in patient numbers

One will come to terms with the virus

more digital offers

as before

increased attention to the individual person (eg some were able to retire earlier - which is sometimes surprisingly good for them!); Promotion of digital media (video visit)

idem

more digital

regular supply is slowly getting underway

Reopening of the diagnostic possibilities

fewer offers

Group offers, easier access to outpatient diagnostics and treatment

not yet predictable

improvement

Easier access, but probably Reduced offers (financial effects on health care will be delayed, but relevant)

Increase in service request

Forced digitization for the purpose of generating more and more data, less comprehensive approaches

What more would you like to see done?

Increase salaries for direct support staff

Appropriate Training for Staff

More studies to evaluate appropriate situations for telehealth vs. face-to-face care

More inclusion of IDD population. Increased funding. Easier access to PPE in community setting.

More study and standardization of service delivery options which have emerged during the pandemic

beginning to remove restrictions so programs can reopen safely

funding support

More certainty on how telehealth will be supported post COVID

INCREASED services post pandemic, increased funding for mental health care.

Increases for essential workers,

Less reliance on virtual consultations.

more vaccination availability. Extremely hard to get vaccinated in our area at this time due to lack of supplies.

More outreach to families, screening for social determinants affecting health, telehealth compensated by insurers

More internet access

more population based care in our state operated services

Better recognition by the government of the risk inherent in the IDD population.

more community supports and consistent staff

I don't know. I practice in several regions; some have risen to the challenge in heroic ways. Others have practiced worsened health care access discrimination against persons with NDD and caused unnecessary deaths

Additional COVID support/therapy groups to incorporate socialization

x

Development of tele-psychiatry

Make things safe so people are more relaxed and feel protected

More virtual

more telehealth and continued team meetings on zoom

more assessment of the validity /reliability of on line assessment tools

Better housing, better choices for housing Paid jobs of the population Vocational opportunities that see them engaged in meaningful activity after graduation Better training of staff and and accreditation of homes Better trading of paraprofessionals so all can care for PwIDD More collaborative teams at the community level For mental heaths and BTC A lifespan approach that follows PWIDD through early years , school, vocation and frailty. This would recognize developmental delay and maximize learning opportunities when the person is ready. Standard expectations for periodic physical exams. Deprescribing programs that include PWIDD

increased training of staff in virtual care and caring during pandemic.

Counselling services for adults with IDD

Better integrated services so people don't end up "agency-hopping" so much and taking up spots on multiple waitlists

Better recognition for work done with this population and their caregivers; better attention to their mental and physical health needs; earlier vaccination.

studies on the impact of these changes

more use of behavioural analysis

more in person service, expanded services to manage backlog

prioritization of people with IDD and their carers for PPE, IPAC, testing and vaccination

Virtual visits/Evidence based medicine.

I need to keep up to date on the skill to provide better care.

focus on equitable care for people with neurodevelopmental disorders

Respite and program access for children is highly needed; caregiver burden has really peaked for many of my families this spring/summer

Continued support for virtual appointments.

ALLOWING ADULTS TO GET BACK INTO COMMUNITY ACTIVITIES

Developmental and other government services to step up to provide individualized supports, health/ mental health care and respond to urgent needs

Bureaucrats more sensitised to nature of disabilities and practical implications.

Evaluate virtual care practices

better financing of pandemic-related failures, more work, measures

better ethical hierarchy between somatic and mental health

more outreach work with appropriate protective measures in patients living alone

n / a

comprehensive digital equipment

digital networking with patients and relatives, video microanalyses, travel routes for the outreach work are adequately taken into account in the reimbursement of costs

Introduce / continue contact-related offers as soon as possible

more exchange in the specialist committees

Better communication between the health authorities and the hospital - especially when it comes to quarantine patients, or the isolation obligations of mentally impaired, restless, disoriented patients.

Individual decisions, for example, patients are refused to bring an accompanying person with them, and hospital stays or outpatient measures are then frustratedly terminated

Restoring previous tried and tested structures

Equality for all patient groups

It's going well with us.

Vaccinations of people with neurodevelop-mental disorders and their caregivers

Transparency, covid test, proof of endurance immunity (with / without vaccination).

Financing of telemedicine

Improved access to vaccination and testing against Covid for those affected and carers

Professionally qualified staff in the facilities

more vaccination offers, less bureaucracy

It is difficult to get an appointment for a corona test (after quarantine) at home for patients who are not very mobile.

Optimization of digital offers

I do not know

Better financing in hospitals for patients with neurodevelopmental disorders

Relief in the event of staff shortages, simplification of refinancing

exchange

Frequent tests to provide the patient with adequate social contacts again.

Advancing digitization in medicine too

More cooperation, closer cooperation in outpatient and inpatient care

significantly faster vaccination!

better vaccination practice, clearer lines, more digitization

more proportionality, inclusion of different opinions

Everyone who has something to say should listen to those who work with the people and not decide something just because there is more money.

Have you developed resources or innovations to aid the current pandemic? If so, what? (e.g. leaflets, crisis pathways etc)

Presentations, leaflets

Webex and Zoom classes

Use of telehealth

Video training, Covid information dashboard for all employees, screening tools, vaccine clinics, Covid Protocols.

Pamphlets

Yes, FAQs and new service options

No

several resources, social stories, webinars

email, resource materials, phone call

Teledentistry

No - use variety of resources from AAP, Fragile X groups, Autism Speaks, etc

Urgent Care telemedicine services

Partnering with behavoiral support entities

No.

Plain language info re: COVID, information from Family Voices on using Telehealth, decision support tools about pros/cons of child's return to school, info on state resources regarding paying family members as trained care-givers

Yes, resources for group therapy by tele health

are system has developed a great number of protocols to aid direct service staff in understanding how to respond to particular levels of symptoms. are division has also developed a series of briefings for supervisors in managers about the current status of the pandemic, and how to apply this knowledge in day-to-day work. Recordings and power points been developed

Telehealth process

Videos, written summaries, verbal discussion points for receptionists and providers to convey.

leaflets and crisis pathways

training videos, written material, live(zoom) presentations, increased direct availability for questions, privately funded COVID-19 testing for persons with NDD and their carers

Safe guards-COVID resources and coping strategies

x

Research and publications on mental health outcomes during the pandemic

posters, teachings, support networks

no

Online resources

list of counselors who do online counseling

no

yes - mainly written resources

We have made available resources to explain Covid and how to keep safe and prevent spread. We have collaborated to make pamphlets for understanding and getting the vaccine. We have advocated for priority of vaccine for this population and those that care for them.

infographs for service users and carers, developed new tool-kits to manage COVID-19 related situations such as hospitalization, implementation of public health measures such as masks etc., developed training programs related to supporting mental health needs of people with NDD at the time of COVID-19.

leaflets

yes.

Crises management/reaching out to families.

Partially- have been involved in educational videos around covid-19 and covid-19 vaccines for patients with NDD

we have program manager to address these issues.

Yes. guidelines, inforgraphs, webinars and capacity buildig sessions in the community

handouts or emails with links to local resources and free online resources.

Refer to HCARDD and DDPCP

No

yes, seizures emergency and urgent phone calls

Leaflets

Yes, virtual care groups for patients and families

Leaflets, video sessions for groups

no

no

Triage sheet on admission

Information material, contact points, hotline

Information letters, compilations of advice options

Telephone consultation hours extended

additional use of video telephony

Working with video microanalysis, digital work, telephoning, video conferencing, outreach work such as going to school, outreach work also for crisis interventions, for example in the children's hospital

Crisis intervention plans, clarification in simple language

Information from the ministry of health

Brochures, group therapies on the ward on topics related to the pandemic

Individual medicine cabinet for the symptomatic treatment of CoViD

simple language (Metacom symbols)

Telemedicine

infection protection was in the focus (successful), adaptation of the therapy offers still necessary (still considerable deficits)

On line treatment.

COVID questionnaires

Personal information, homepage

Implementation of the measures through one-way systems to protect patients and staff

especially for deaf people

Newsletters, checklists,

regular corona testing of the team

brochures

Crisis plans

Flyer

Simple language, posters, brochures, videos

Yes

Brochures,

Crisis paths, test-and-trace strategy across the clinic.

Information sheets on transmission routes, illness, hygiene rules and vaccination in supportive communication and plain language.

Brochures, mail newsletters

no, benefited from the cooperation with KEH-Hospital in Berlin

Crisis paths

We have a small section on the intranet in easy language, but only reaches a fraction of the patients!

share point

If you have time, please give a short summary of your experience in providing care during the pandemic and any further comments

Administration negate the concept that staff is also stressed and need more flexibility

Learned to implement telehealth, working on using it wisely, shouldering an increase in proportion of care that is required of me without others on health team in telehealth.

The administrative workload has significantly increased for all in our agency. Obtaining the necessary PPE has gotten better, but regulations requiring things like respirators have left us out of compliance because we cannot source adequate materials. Communicating remotely has created informational barriers as emails and other forms of remote communication have become so numerous that identifying what is important has become a challenge. Boredom among IDD clients has cause increases in behavior and significant weight gain among many in the population.

Highs and lows; some people have done better with telehealth than I thought likely while others have struggled terribly. Working all the time is how it feels, not much rest

Emotionally and physically exhausting

Dentistry was deemed non essential from March until June , countless teeth were unnecessarily extracted because aerosolized procedures were not allowed. it was one of the worst times in my career . I still cannot believe that ai was not able to provide care .

Uncertainty and tons of regulation changes in the midst of pandemic from State adminsitrative agencies

Relatively high rate of Outpatient Appointment cancellations because of patients' being in isolation. Some difficulty in understanding patients because of mask. I should add that in my country, Israel, the Crisis 80% over (for the moment) , And my answers above relate to the present phase rather than at peak time, when Some families were worried about coming to the hospital or just assumed that my service is closed.

Care is difficult for the IDD population due to the lack of socialization with peers and family. More disruptive behaviors displayed and more need for psychological support has been needed. Population has also been much more sedentary and lacks motivation to do more activity when being isolated at home.

We modified our schedule: staggered arrivals and dismissals...this was a BIG disruption in how we typically schedule; we recently resumed our previous schedule plans/ arrival times and I am so thankful for that More confirmation calls, increased number of mix ups as far as consent, seems to be higher amount of staff turn over in facilities that bring out patients to the Center

our division encountered …REDACTED FOR ANONYMITY… has led me to supervise and consult in regard to a wide variety of new challenges, including getting people tested and getting them treated appropriately. We have a great cadre of RNs who have helped make this work very well for the most part. We have just completed the 2nd round of clinics to be sure that all who want the vaccine can have it. On a day-to-day basis, the biggest problems for the individuals we serve has been related to being locked down into the homes and not being able to get out to day programs, work settings, or just recreational activities. This does seem to have increased levels of anxiety and depression, and in some cases has led to more episodes of aggression. More broadly speaking, people with NDD in the general community have had a great difficulty in obtaining vaccination because our system has not recognized them as being in a more vulnerable population.

Ethics- Double edged sword. On one hand it has greatly accelerated the adoption of telehealth. However, there are ethical issues specific to the IDD population that are lost with the desperate need to have access during the pandemic. My concern is that people will not go back and address these issues once telehealth is more “optional” as a delivery modality.

this has been a challenging time to adapt to our new way of providing services and clients being able to access virtual service

As a physician i have had to stretch into areas of practice which were not part of my original job description. Fortunately in the USA extra medical training from credible sources (Harvard, Mayo, others) has been available virtually to help guide this expanded scope of practice. I also needed to make myself available 24-7 to individuals with NDD and the people who support them

x

-

I wanted to see as many patients in the clinic live and demanded that the clinic allow it. I was granted permission and I have to say I am one of the few as many colleagues have not returned to clinic and work remotely. For me it does not work and I need the in-person contact to have rapport with patients and caregivers.

Increased virtual telehealth and increased online resources

I have enjoyed when group home staff are able to keep pharmacotherapy appointments going through the pandemic by helping clients get on zoom. I have liked the new access to zoom team meetings to discuss problem behavior as a team.

I have found virtual assessments more useful than i would ever have imagined . I communicate with patients via email a lot more than before but i worry about missing emails . My technology skills have improved . I miss day to day interactions with colleagues I do a lot more clerical work . I find virtual work more tiring . Not sure if this is because my "screen time" is hugely increased I have developed wrist pain associated with increased use of keyboard

It about time, time to advocate, time to look for resources that are outside the box, time to support family and caregivers, time to keep Up on what is going on in the country and world so you can present it to stakeholders and do all your other usual, teaching, clinic and research duties. I cannot stress enough, primary care providers are often called to be jacks of all traits when providing care for this population, OT,PT, BT, MSw, admin staff, something has to give.

social service supports always been terrible and just worse

we are hanging there but with chronic fatigue

I find that the pandemic was positive in the way that it encouraged the quick conversion to telehealth. I find a fair number of patients have benefits from us being able to provide virtual care. I think it has made attending appointments more convenient and less stressful for care-givers. This has been an unanticipated benefit.

Missed on week March 2020 and then pivoted to clinics from home on telephone, for several months until was given access to video platform; even with this, staff and families struggle to connect; difficult to do full assessment if cannot see patients; comorbidities increasing; cannot recommend non-pharm interventions, eg walking due to severe lock downs in MCSS; great collaboration with many PCP's; vaccine roll out to IDD adults a disaster and tragically delayed!

Challenging when no programs and caregiver burnout. No where to go. haven't used medication but not sure that was helpful either. Lack of professional support services and day programs restricted for longer than daycares, for example. Seemed excessive.

The isolated community in which I live and work has had almost no cases of COVID, so that has made clinical care easier.

As a long time care provider I have good connection with patients,they are not anxious when they see me and I make sure to make them feel comfortable.

I have been involved in providing care for IDD patients for long time and continue during the pandemic also.

I have found myself taking on more medication monitoring and titration that I did prior to the pandemic as a sub-specialist. Partly as prescribing feels that it has increased and partly because access to primary care was variable for families. This has meant more clinical visits fit in around my other obligations as an academic physician. compounding stress due to conflict between academic 'deliverables' and clinical care as an early career physician. Providing closer follow up was one way I tried to help families stay connected/safe especially as we were augmenting treatment or if there were social concerns. I found I could not rely on the community agencies as I would have in the past to do some of this monitoring as it 1. became harder to locate workers (playing phone tag as people worked remotely), 2. service capacity was reduced and 3. kids weren't in school or going to childcare where other supervision was occurring. I don't know if this is the correct approach. Doing more doesn't necessarily mean better care. and it's a fine line between adding stress to parents with more visits and providing them that support. More recently I have had families vent their stress with the system during their appointments; some have been very angry and sad. They feel their children with ID/NDD have been 'forgotten' and left out. The lack of redundancy in our system…REDACTED FOR ANONYMITY… for school age kids especially, is a problem. If kids aren't in school they're not accessing their PT/OT/SLP, unless parents are savvy and know this is what their child is eligible for. I've had some kids go the entire year without a check in from SLP and their communication delays and behaviour reflect this.

ONLINE AND PHONE ASSESSMENTS DIFFICULT; MANY ADULTS CANNOT PARTICIPATE; CANNOT DO FULL MSE; LACK OF JOB SATISFACTION AND INTERPERSONAL CONNECTION MAKES WORK LESS FULFILLING.

People with IDD in SiL less supported and those in congregate settings held up in homes without the stimulation and engagement they need

There is a spirit of meanness in bureaucrats so it is hard to get funding and support

private and professional constriction with limited availability of resources; Limitation to the essentials with positive and negative effects!

My observation is that some (especially autistic) people benefit from the lockdown (less overwhelming), but many suffer from the changed daily routine.

Great effort through constant tension regarding the correctness and security of one's own approach, great emotional burden in the experience of the stresses of patient families

In the…REDACTED FOR ANONYMITY…there are more time resources available compared to everyday outpatient clinical work, more opportunities to work independently, to respond to the needs of the individual patient, digitally, by telephone, video, ..

Outreach work as added value in diagnostics and treatment. Liaison and consultation services, regular low-threshold advisory services in the institutions. Unfortunately, we are a very small team and professional colleagues still have reservations about the persons with neurodevelopmental disorders, even though they provide us with such valuable professional experience and teach us a lot.

We had to structure the work more, implement the hygiene rules in everyday life, work with protective equipment (which is sometimes tiring) PS: the questions are sometimes misleading!

The so-called pandemic protection measures represented a much greater (in my opinion: too great) burden than the actual threat from the disease itself. (Small note: I think this survey is good and important and I am happy to participate. Due to the necessity to give the Email, however, suffers from anonymity a little ...)

The Swiss vaccination strategy does not take this vulnerable patient group into account. however, the medical care took place within the usual framework.

Partly inhuman regulations for certain residents, since no exceptions were allowed at the beginning, especially with regard to isolation, generation of irrational fears, the development of compulsions, tics ... Deep rifts between colleagues who were completely corona compliant and others who questioned certain measures and were critical and demanded more creative solutions. Incredibly one-sided reporting, complete lack of information on vaccinations before the vaccinations and pressure to have them carried out "out of solidarity" or to process residents / relatives so that they can do it

Initially massive symptoms of stress / secondary trauma due to a lack of clinical information by the specialist societies, due to dictatorial interventions by the authorities in everyday practice and circumvention of general practitioner competence by institutions in cooperation with the cantonal medical service

many relatives and institutions were very alone with their people with neurodevelopmental disorders, our outpatient clinic was temporarily closed during lockdown; a lot of telephone contacts, great gratitude

Many appointments have been cancelled because the clients / families / living groups are anxious/insecure. Often the living groups / families do not have good WiFi / PCs for video conferences or they are not willing to do so. Conference calls cannot be billed well enough.

There is generally a lack of transparency in relation to pandemics. No measures to strengthen and optimize the immune system are recommended.

Missed appointments due to COVID infections in residential facilities. Reduced reception capacities in hospitals.

More services because staff are partly sick or in quarantine. Overload (in terms of to much stress)

step in more, few staff due to quarantine, sick leave, chaos due to pandemic measures,

The extremely bad cooperation with the overwhelmed heads of the residential homes was nasty, exhausing

too slow reaction of the government, authorities, company, care management level,

Significantly increased stress and more work, e.g. due to the absence of colleagues who fell ill or had to be in quarantine because family members were sick or because the children in need of care had to be in quarantine.

The daily changes to the organization and hygiene regulations, the communication with the health authorities, the unequal handling of quarantine regulations (often a lack of leeway here), a lack of exchange within the team (no more team meetings due to hygiene standards, or if so, then only in small groups) were exhausting), reduced medical exchange, hardly any further training offers in 2020

not specified

Delay of important diagnostic measures by stopping admission to hospital

The pandemic tied up an excessive amount of time, which was therefore too short for other necessary topics. Due to the contact restrictions but also due to the shortage of staff, patients were admitted with some delay

Dealing with testing and vaccination, observation of symptoms

I tried to continue the quality of our outpatient clinic work unaffected, that was challenging enough!

overall a great psychological burden for everyone

It was very stressful because I had the role of corona counseling (link between health authorities, carers, carers and relatives and vaccination coordination. Again and again new regulations that had to be adapted and implemented. Less in the medical practice, but more within the facility and the whole Vaccination coordination that was left to the facilities to look for mobile vaccination teams and to plan and carry out the vaccinations in a large organization.

Surprisingly uncomplicated integration of the processes in the very well working facilities. The changing hygiene requirements by politics have made it a bit confusing. The facilities each have requirements for hygiene (online advice with video tool, advice only 1: 1 with specialists, normal advice. Further different protective measures: Large room without mask, normal examination room with mask, etc. Very noticeable: People with emotional reference ages below 4 years (SEED 1-3) have emotionally benefited more from the abolition of the 2-milieu principle and showed fewer abnormalities/challenging behaviours. Pat with higher levels of emot. development levels tend to show more behavior problems due to the hospitalization. (With the now longer duration of the restrictions, however, this effect is blurred towards hospitalization signs.

Frighteningly inhuman conditions, especially in the inpatient area and in the elderly, due to refusal of personal attention, deterioration in medical care, excessive reactions in panic mode, little questioning thinking, little forward thinking, sometimes a lack of proportionality, a lot of system pressure to suppress dissenting opinions Emotional instead of factual debates, the patient not as the person to be treated, but as the one endangering the doctor, that is particularly frightening for me.

END OF RAW DATA
