# Supplementary figures and images for: International clinician perspectives on pandemic-associated stress in supporting people with intellectual and developmental disabilities
Source: BJPsych Open. 2022 Apr 18;8(3):e84. doi: 10.1192/bjo.2022.49 (PMC9059730; doi:10.1192/bjo.2022.49)

**Supplementary material Survey (English version)**


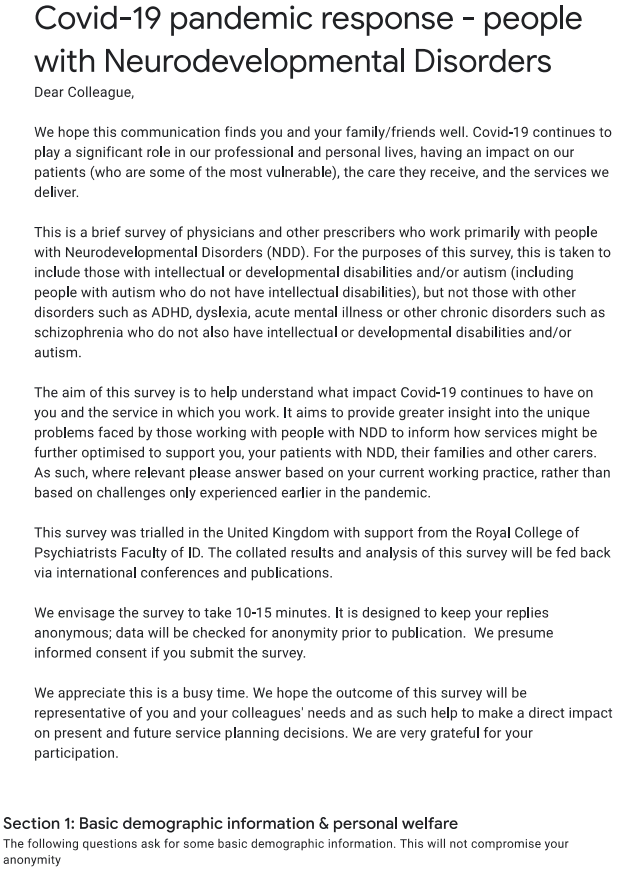


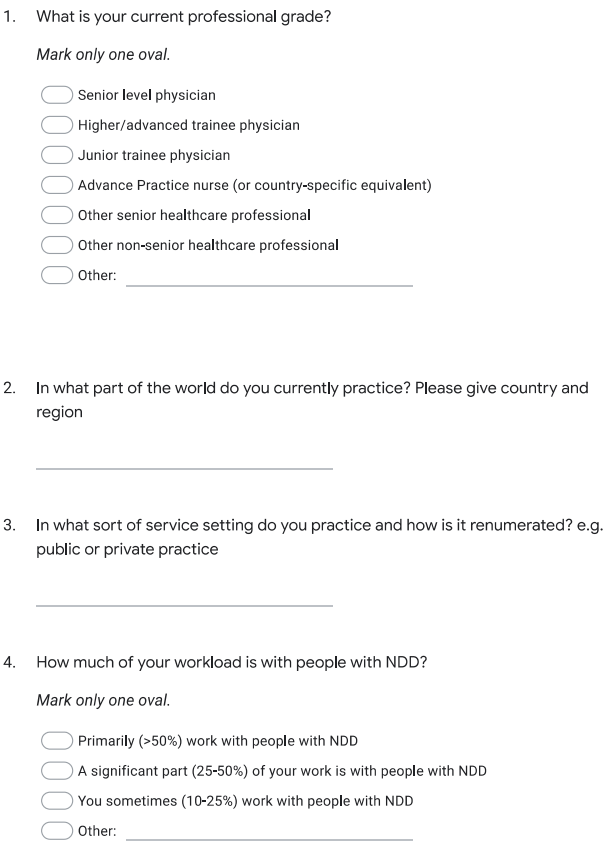


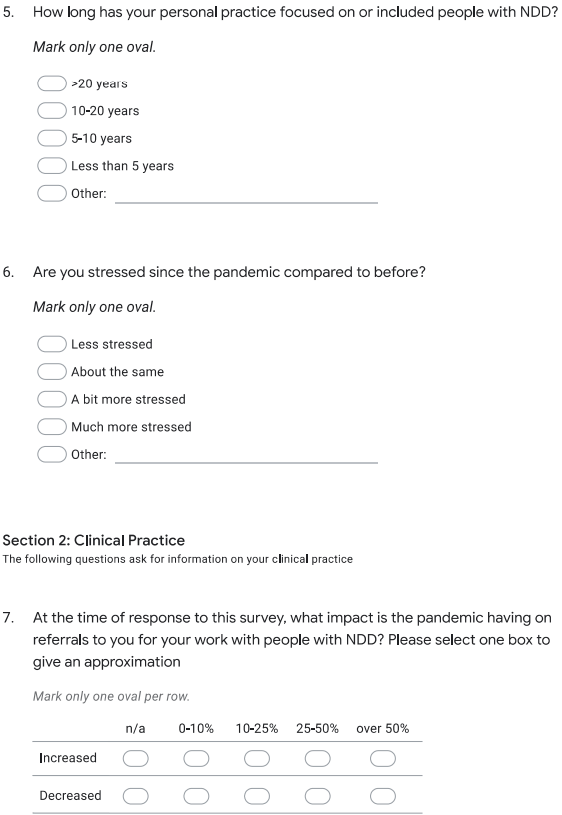


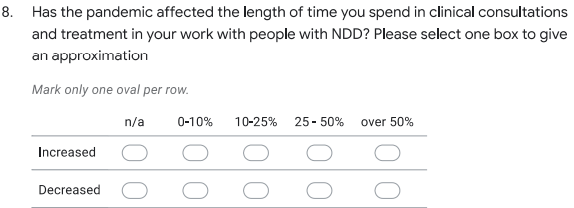


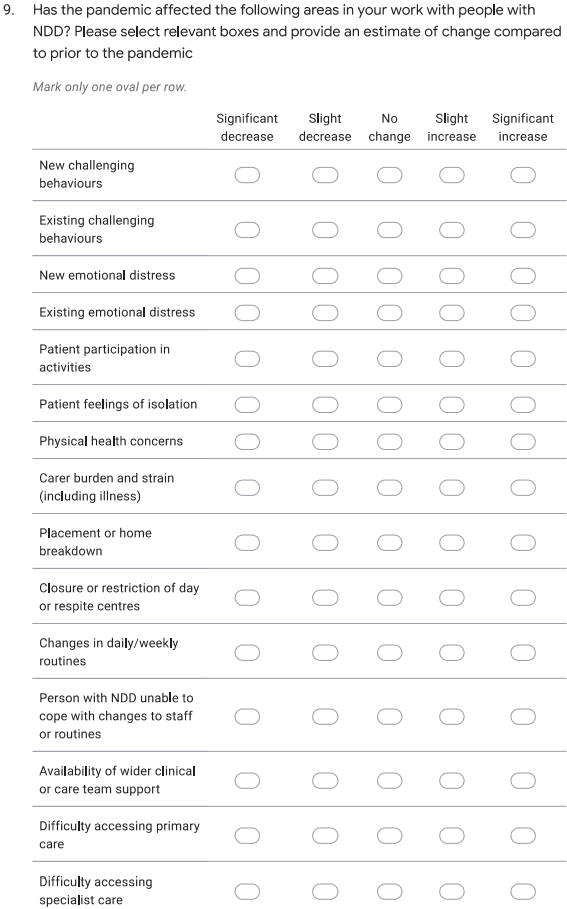


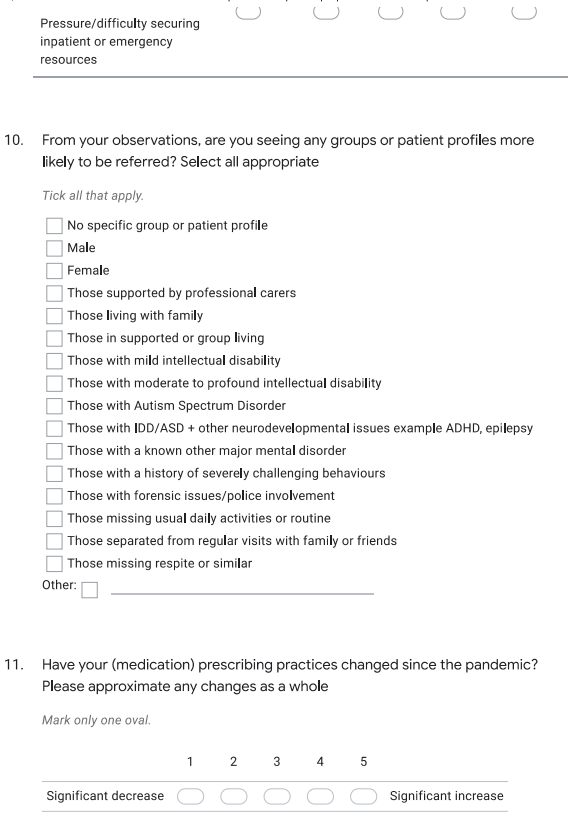


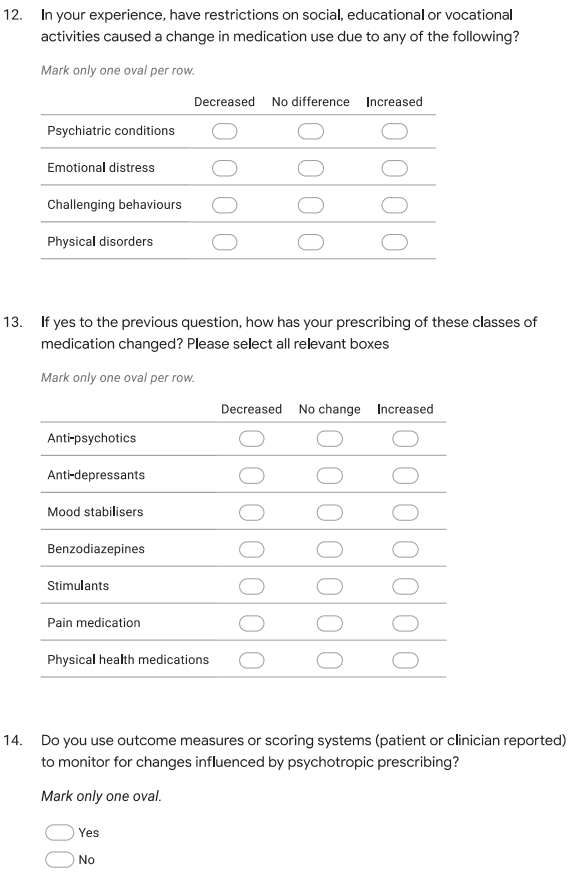


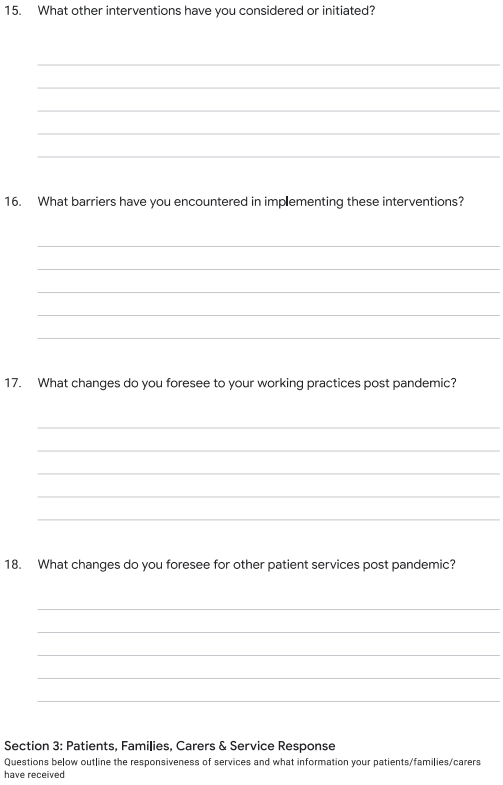


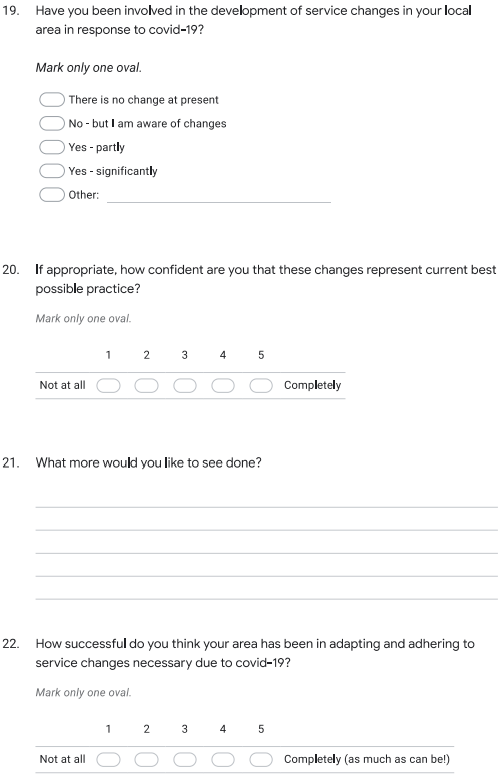


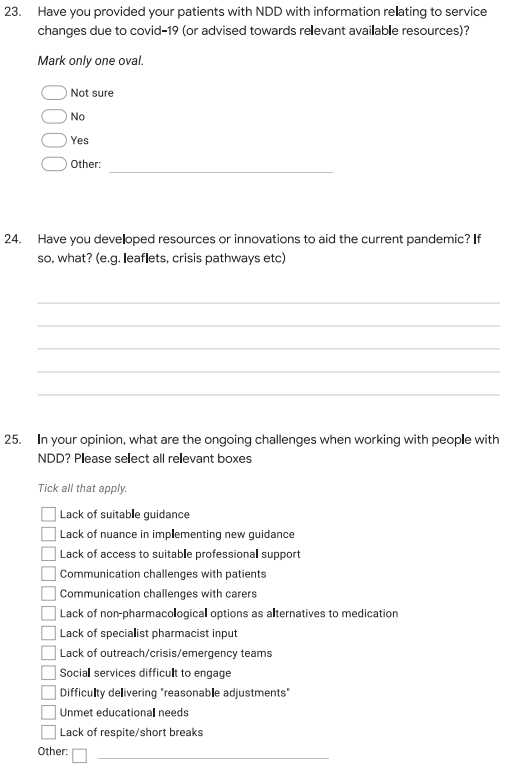


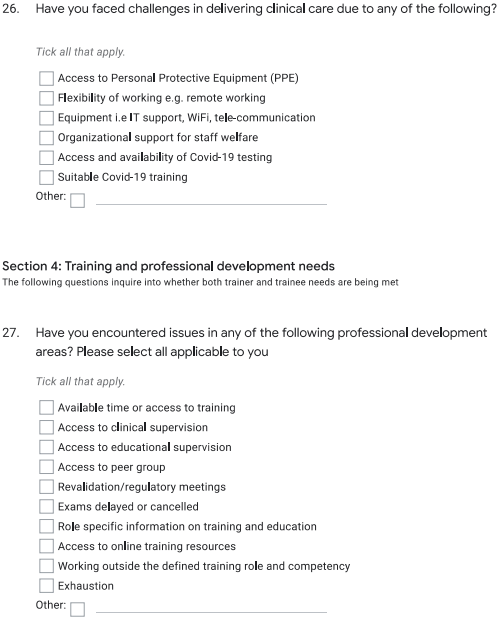


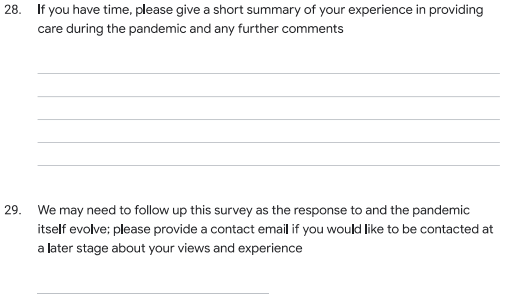

Supplement: Supplementary file 1 [file bjosup.zip › S2056472422000497sup001.docx]
